# Peace and the Profession

**Published:** 1919-07-12

**Authors:** 


### PEACE AND THE PROFESSION.

FOR the medical, as for every other profession in the country, the fateful period between the signing of the Armistice and peace has been filled with the greatest uncertainty for the future—uncertainty, not so much for the prospects of scientific and medical advance as a whole, because these national necessities appear, if one can judge from public announcements and the Press, to be receiving more than a little consideration. Rather do we note an undisguised anxiety of the individual on matters of welfare and livelihood, and this feeling is strongly reflected in regrettable and bitter divisions in the medical camp itself.

There are numberless possible causes which may, in a doctor, give rise to a sense of honestly felt worry or grievance, but at the moment probably the commonest and most potent of all comes from difficulties of a pecuniary nature. Take what branch we will, the cost of professional existence has increased to a degree comparable with that experienced in any other sphere of life, and, although there are now a number of factors tending towards remedy, the readjustment is as yet far from complete.

Raising of fees has been an obvious measure for those men already well established in private practice, and was extensively carried out in this and most of the Allied countries. The Americans quickly and thoroughly reorganised their scale, but with our, in these matters still conservative, country it has not been so generally practicable as many of the laity believe. In relatively few instances has the result been commensurate with the doctor's justifiable needs. Also we have an announcement by Major Astor that the amount to be provided from the Exchequer during the present financial year for extra remuneration in the nature of a war bonus for panel practitioners is £300,000, and the more recent details of this scheme provide a sign of Governmental recognition, even though benefit to the individual is but moderate. In some quarters, too, notably the Services, there is a definite rise in the rate of medical pay; but, nevertheless, one has but to scan the advertisement columns of the medical journals to realise that this tendency is far from general, and that the overwhelming majority of paid medical posts show but poor increase in remuneration on the pre-war rates.

Now the country has applauded and welcomed the principle of team work, the scheme for men to work unhindered on innumerable problems with which the intricacies of disease present us. Some must work among the people, some in the laboratory; some must specialise and become expert in their specialty, some by mentality are more fitted for general lines. But every one of them, to produce his best, efficiently to play his part in the coming health campaign, must have reasonable freedom from the worries of domestic ways and means.

It would be absurd to suggest that any reasonable plan can make every doctor in the country free from need for domestic forethought, but, nevertheless, it may be pointed out that nowadays on every hand are instances of men being asked to do, and actually doing, work of urgent medical importance for a return ridiculously below the minimum living wage due to a professional man with a home. Among the many coincident facts of this situation, two are particularly noticeable. The first is that a number of these men are able to give but a part of their best to true advancement of medical knowledge, the rest being spent in search of the very necessary guinea. The second is that at present a qualification of a man deemed fit for opportunity to develop a high degree of specialisation or post graduate knowledge—the only means, in the present wide medical fields by which rapid progress in research can be made—is often that he must possess private means. These are but two instances taken from the curiously disunited medical and institutional organisation which exists at the moment. They may not apply to every part of the country, but instances of these or similar causes of inefficiency can be found galore, and against them vigorous protest is requisite.

We need research; we have never been in a position more favourable to initiate its development; and we must realise that, for the ideal, every medical man, however moderate his attainments, either as examinee or practitioner, must be given opportunity to play his part whole-heartedly. Whether his income be derived through a national medical service, as a salary from an institution, or as a grant for some special work, then in proportion to the percentage of his time demanded he should receive at least a professional living wage. The subject is complex, innumerable side arguments present themselves, but the fact remains that, under post-war conditions, an enormous number of salaried medical appointments are ridiculously underpaid if the time and energy of the doctor are seriously intended for efficient and exclusive concentration upon the valuable work to which these posts give him access.

